# The Role of Glucocorticoids in Breast Cancer Therapy

**DOI:** 10.3390/curroncol30010024

**Published:** 2022-12-25

**Authors:** Irma B. Mitre-Aguilar, Daniel Moreno-Mitre, Jorge Melendez-Zajgla, Vilma Maldonado, Nadia J. Jacobo-Herrera, Victoria Ramirez-Gonzalez, Gretel Mendoza-Almanza

**Affiliations:** 1 Unidad de Bioquimica, Instituto Nacional de Ciencias Medicas y Nutricion Salvador Zubiran (INCMNSZ), Mexico City 14080, Mexico; 2 Centro de Desarrollo de Destrezas Médicas (CEDDEM), Instituto Nacional de Ciencias Medicas y Nutricion Salvador Zubiran (INCMNSZ), Mexico City 14080, Mexico; 3 Laboratorio de Genomica Funcional del Cancer, Instituto Nacional de Medicina Genomica (INMEGEN), Mexico City 14610, Mexico; 4 Laboratorio de Epigenetica, Instituto Nacional de Medicina Genomica (INMEGEN), Mexico City 14610, Mexico; 5 Departamento de Cirugía-Experimental, Instituto Nacional de Ciencias Medicas y Nutricion Salvador Zubiran (INCMNSZ), Mexico City 14080, Mexico; 6 Consejo Nacional de Ciencia y Tecnología (CONACYT), Laboratorio de Epigenetica, Instituto Nacional de Medicina Genomica (INMEGEN), Mexico City 14610, Mexico

**Keywords:** glucocorticoids, glucocorticoid receptor, breast cancer, progression, triple-negative breast cancer

## Abstract

Glucocorticoids (GCs) are anti-inflammatory and immunosuppressive steroid molecules secreted by the adrenal gland and regulated by the hypothalamic–pituitary–adrenal (HPA) axis. GCs present a circadian release pattern under normal conditions; they increase their release under stress conditions. Their mechanism of action can be via the receptor-independent or receptor-dependent pathway. The receptor-dependent pathway translocates to the nucleus, where the ligand-receptor complex binds to specific sequences in the DNA to modulate the transcription of specific genes. The glucocorticoid receptor (GR) and its endogenous ligand cortisol (CORT) in humans, and corticosterone in rodents or its exogenous ligand, dexamethasone (DEX), have been extensively studied in breast cancer. Its clinical utility in oncology has mainly focused on using DEX as an antiemetic to prevent chemotherapy-induced nausea and vomiting. In this review, we compile the results reported in the literature in recent years, highlighting current trends and unresolved controversies in this field. Specifically, in breast cancer, GR is considered a marker of poor prognosis, and a therapeutic target for the triple-negative breast cancer (TNBC) subtype, and efforts are being made to develop better GR antagonists with fewer side effects. It is necessary to know the type of breast cancer to differentiate the treatment for estrogen receptor (ER)-positive, ER-negative, and TNBC, to implement therapies that include the use of GCs.

## 1. Introduction

Glucocorticoids (GCs) are steroid hormones that function as anti-inflammatory and immunosuppressive agents; they have been used since the 1940s, primarily in the treatment of autoimmune diseases and inflammatory disorders, to inhibit organ transplant rejection, and to minimize the side effects of oncologic chemotherapy [1,2].

The natural GC is cortisol in humans, and corticosterone in rodents. CORT is secreted by the adrenal gland and is regulated by the HPA axis. The hypothalamus secretes corticotropin-releasing hormone (CRH), which stimulates the production of adrenocorticotropic hormone (ACTH) from the pituitary gland, which promotes the release of CORT in the adrenal gland [3,4].

The main function of GC is in response to stress stimuli, whether physical or emotional. GCs are involved in the regulation of blood pressure, inflammation, immunosuppression, neural plasticity, body temperature, the control of food intake, and sleep modulation [5,6].

Free CORT enters the cell via passive diffusion, mainly because it is a fat-soluble molecule [7]. GC molecules circulate in the blood, bound to corticosteroid-binding globulin (CBG), also called transcortin, and can be released by the enzyme elastase [8,9]. Another small fraction of CORT is bound to albumin (approximately 15%), and a smaller fraction of 5% is free; this is called the bioavailable fraction.

There are several synthetic GCs whose chemical structures contain targeted changes to optimize their activities, such as an increase in their liposolubility, facilitating their introduction into cells, or an increase in their bioavailability [10]. DEX is a synthetic GC that is widely used in the clinic, and its anti-inflammatory potency is 30 times higher than CORT [11].

Human CORT levels are regulated in a tissue-specific manner by 11β-hydroxysteroid dehydrogenase (11β-HSD) 1 and 2; 11β-HSD1 modifies cortisone that is inactive to CORT to induce multiple signaling cascades, and 11β-HSD2 maintains inactive CORT via the reverse process [12].

## 2. Genomic Effects of Glucocorticoids

At the cellular level, GCs have two main effects, genomic and non-genomic. Genomic effects occur through GR, encoded by the NR3C1 gene located at the 5q31.3 locus, and produce two proteins via alternative processing, GRα and GRβ, with 777 and 742 amino acids, respectively. However, each isoform has at least eight subvariants, which promotes multiple responses [3,13,14].

The genomic mechanism of action of GC is given by binding the ligand to its GR located in the cytoplasm in an inactive conformation bound to multiple protein complexes that include heat shock proteins such as Hsp90, Hsp70, immunophilin p59, phosphoprotein p23, and cytokines [15,16].

When GCs bind to their GRs, they cause their activation and translocation as a complex to the nucleus to carry out the transcription of various genes by binding to specific sequences in the DNA. These sequences are in the promoters of target genes termed glucocorticoid response elements (GREs), thus regulating the transcription of specific genes such as serum/glucocorticoid regulated kinase 1 (SGK1), mitogen-activated protein kinase 1 (MAPK1), phosphoinositide 3-kinase (PI3K), and glucocorticoid-induced leucine zipper (GILZ) [3]. The sequences in DNA to which the GR binds as a dimer are 15 base pair sequences 5′-AGAACAnnnTGTTCT-3′ [17,18], and the negative GRE consensus sequence has been described by Hudson et al. as 5′-CGCCTCCnGGAGAGCT-3′ [19].

GR is expressed in all mammalian cells and can regulate gene expression via a transrepressor mechanism by recruiting other transcriptional factors such as Activator Protein 1 (AP-1) and Nuclear Factor-kappa B (NFκB) to repress inflammation-associated gene expression [3,20].

It is well documented that GR can undergo six post-translational modifications: phosphorylation, SUMOylation, ubiquitination, oxidation, and acetylation in its different structural domains. These modifications produce changes in the expression of target genes; in binding with other proteins, compositional changes, the capacity of nuclear exportation, and biological processes in which the GR intervenes are affected as a cell cycle, or as apoptosis. Phosphorylation is performed by enzymes such as p38 mitogen-activated protein kinases (p38 MAPK), glycogen synthase kinase 3-beta (GSK3B), MAPK, cyclin-dependent kinases (CDKs), c-Jun N-terminal kinases (JNKs), and serine/threonine-protein kinases (AKT1). Dephosphorylation is involved with LBD-linked protein phosphatases as Ser/Thr protein phosphatase type 5 (PP5).

The acetylation of GR modifies its binding to DNA; this modification involves acetylases/deacetylases and the complex Circadian Locomotor Output Cycles Kaput (CLOCK)/ Basic Helix-Loop-Helix ARNT Like 1 (BMAL1). Ubiquitination leads to its GR degradation after binding to DNA, this process involves the system E2/E3 ubiquitin enzymes. 

SUMOylation occurs in a residue in the ligand binding domain via the SUMO-1-conjugating E2 (UBC9); this modification modifies its ability to bind to other molecules such as proteins or DNA, their location, and their activities in transcribing genes. Finally, oxidation modulates the overall activity of GR. GR post-translational modifications are summarized in (Table 1) [4,21,22,23].

When CORT or DEX binds to GR, anti-inflammatory and immunosuppressive processes, and biological processes such as apoptosis, proliferation, and angiogenesis are activated. For this reason, they have been used to treat diseases such as asthma, arthritis, autoimmune diseases, and cancer [5].

## 3. Non-Genomic Effects of Glucocorticoids

**Figure 1:**
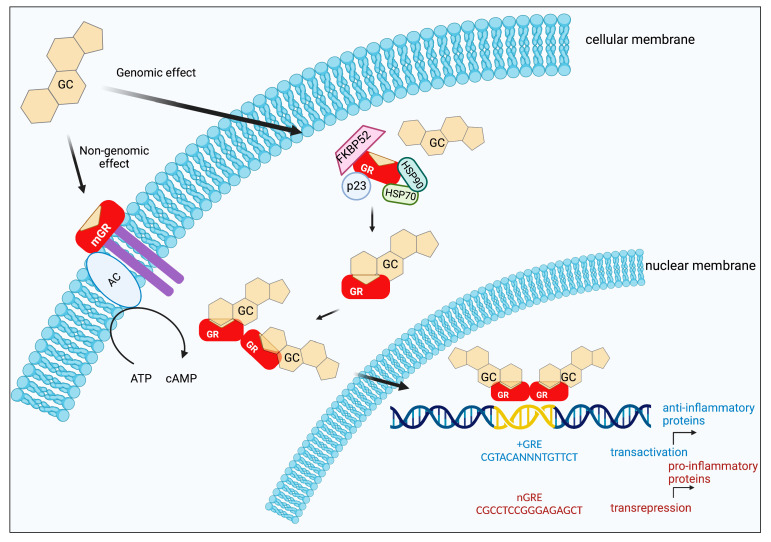
Genomic and Non-genomic effects of GCs. p23: a protein associated with progesterone receptor (23 kDa), HSP70 (70 kDa), and HSP90 (90 kDa): are heat shock proteins, FKBP52: FK506 binding protein (52 kDa), AC: acetylcholine. Created by Biorender.

GCs may exert actions in a non-genomic way; Johnstone et al. mention five requirements for it to be considered a non-genomic mode: being quick to respond within minutes or seconds of exposure to GCs, being insensitive to transcription and translation inhibitors, being insensitive to transcription and translation inhibitors, and being reversible. We can group these non-genomic responses of the GCs into several main modalities that are summarized in (Table 2) and (Figure 1) [24].

It is important to mention that both cytosolic and membrane GRs originate from the NR3C1 gene, although membrane GRs appear to be linked to the expression of exon 1A-containing GR transcripts which produces a receptor with a high molecular weight of 150 kDa [25]. Vernocchi et al. [26] reported membrane-localized GR (mGR) activation by BSA-conjugated cortisol, and in some cells, including a breast cancer cell line mGR, residing in association with caveolin-1, and possibly work via Rho signaling. Matthews et al. [27], using coimmunoprecipitation studies, identified the interaction between GR and caveolin in the lung epithelial cell line A549; this system is also involved in the activation of protein kinase B and produces a modulation in the proliferation capacity of cells.

Several observations suggest that this interaction could be cell- and context-dependent. For example, Spies et al. reported that mGRs are upregulated on monocytes after in vitro stimulation, and in patients with rheumatoid arthritis. The frequency of mGR monocytes in patients did inversely correlate with GCs dosages, and mGRs are not colocalized with caveolin-1 in plasma membrane caveolae [28].

The non-genomic effects of GCs are characterized by the fact that they occur within a short period, within seconds after GC administration. Their mechanism involves cell membrane and membrane-associated protein involvement; when the GC interacts with the cell membrane, there is no nuclear translocation. These non-genomic effects could be mediated mainly by mGR or in the cytoplasm (cGR), where they interact directly with other signaling pathways such as the MAPK pathway, or they directly interact with other molecules; however, this pathway is less frequently explored today. A better understanding of non-genomic pathways may open new perspectives for the development of therapeutic strategies [34,35,36,37].

## 4. Glucocorticoids and the Circadian Cycle

The circadian rhythm is a universal regulatory system that creates daily rhythms over a 24-h period to maintain homeostasis. It uses signals received from the environment, such as light and nutrition, to regulate physiological and metabolic events [38]. Epidemiological and laboratory evidence suggests a link between the circadian clock and cancer [39]. Under normal conditions, the secretion of GC follows a very marked circadian rhythm. However, upon a stress stimulus, this secretion pattern may be lost. Essential circadian rhythm hormones such as melatonin, testosterone, and GC dictate circulating tumor cell generation [40]. Diamantopoulou et al. observed that circulating tumor cells (CTCs) have the capacity to extravasate more during the sleep stage than during the active phase. By performing RNA seq studies, they determined the existence of mitotic genes generated during the sleep stage, in both human and murine models. They found that key circadian rhythm hormones dictate the dynamics of the generation of these CTCs, suggesting the need for time-controlled approaches to the characterization and efficient treatment of breast cancer [41].

Pulses release CORT according to the circadian cycle with a 24-h oscillation, to prepare the body for rest and to enter a sleep state. The levels of adrenaline, noradrenaline, and CORT decrease, causing muscle relaxation and the sensation of tiredness. Additionally, if their blood concentrations increase, they allow the muscles and the brain to be active and alert [42,43]. Then, CORT in humans has its maximum concentration (zenith) upon awakening (7:00 h) and reaches its minimum concentration (nadir) at bedtime (22:00 h) [11]. CORT is an important circadian hormone that regulates the circadian rhythm, and it is known to interfere with the apoptotic effect of chemotherapy and other anticancer agents [44]. Such agents would be more effective if they are administered during the CORT nadir. This fact may modify the efficiency of breast cancer therapy [45,46].

Clock genes have been described to play a pivotal role in disease development due to the intercommunication of the endocrine system and the circadian cycle. Several hormones such as ghrelin, leptin, melatonin, insulin, adiponectin, and CORT are regulated by the circadian cycle. Chronic stress, circadian rhythm alteration, inflammation, metabolic syndrome, obesity, depressive behavior, and major depression, which have been associated with GC effects, have also been linked to accelerated cell proliferation, and the increased risk and progression of breast cancer [47,48,49]. The circadian clock has been determined to be formed under four gene controls called CLOCK, BMAL1, Cryptochrome, and Period. The CLOCK-BMAL1 complex affects up to 10% of all transcription in the circadian cycle. The loss of several tumor suppressor mechanisms caused by an altered circadian rhythm contributes to carcinogenesis. The dysregulated expression of numerous circadian cycle proteins has been found to be associated with poor prognosis and aggressive behavior. Single nucleotide polymorphisms in the Cryptochrome Circadian Regulator 2 (CRY2) and CLOCK genes have also been shown to increase the risk of breast cancer. In the few epidemiological studies conducted, an increased risk of breast cancer has been reported in people who work night shifts [38]. The dysregulation of the circadian clock leads to the inability of the organism to maintain the circadian rhythm of key biological functions, such as metabolism, angiogenesis, immunity, DNA repair, and cell cycle regulation. In cancer, melatonin plays pleiotropic roles and effectively inhibits epithelial-mesenchymal transition (EMT), and thus tumor dissemination and metastasis, through the negative regulation of proinflammatory IL-1β/NF-κB/MMP2/MMP9 pathways, extracellular matrix remodeling, and mesenchymal gene. The dysregulated circadian clocks in tumor cells lead to a dysregulated production of VEGF, generating continuous rather than cyclic stimulation, and growth of the tumor vasculature, which promotes metastasis [50].

Clinical studies have shown that CORT levels could be a valuable marker for predicting survival rates in breast cancer patients. Allende et al. analyzed CORT levels in saliva samples (morning, afternoon, and evening) from 93 patients with recurrent breast cancer and patients with metastasis. The results showed that CORT levels in the morning are similar in patients with cancer and in women without cancer. However, in the afternoon, CORT levels were higher in cancer patients [51].

Abercrombie et al. observed in patients with metastatic breast cancer a decrease in the slope of the diurnal variation of CORT, compared to healthy individuals. They found that metastatic disease in patients with higher severity had higher CORT levels [52].

In contrast, Sullivan et al. found that Balb/c mice carrying breast cancer lack circadian variation in circulating corticosterone levels. These mice exhibit lower corticosterone levels than normal mice at most times of the day. In addition, surgical removal of the tumor results in the recovery of the 24-h rhythmicity of corticosterone levels [43].

Primary, recurrent cancers worldwide are related to endocrine modifications, including breast, uterine, thyroid, prostate, ovarian, and pancreatic [53]. Genomic studies have shown a clear association between the dysregulation of clock genes that regulate the circadian cycle, and cancer development [54,55]. Genetic variants in the clock genes have been found to be associated with breast cancer [49,54].

## 5. Glucocorticoid Effects on Mitochondria

The effects of GCs on mitochondria depend on the dose and time of exposure. GC has generally been detected in different cell types where GC increases mitochondrial biogenesis at low doses or in short exposure times with increases in respiratory chain enzymes [33,56]. In contrast, at high concentrations and/or prolonged times, they cause a decrease in mitochondrial activity and also in membrane potential, causing low ATP production and an increase in reactive oxygen species (ROS) [33].

**Figure 2:**
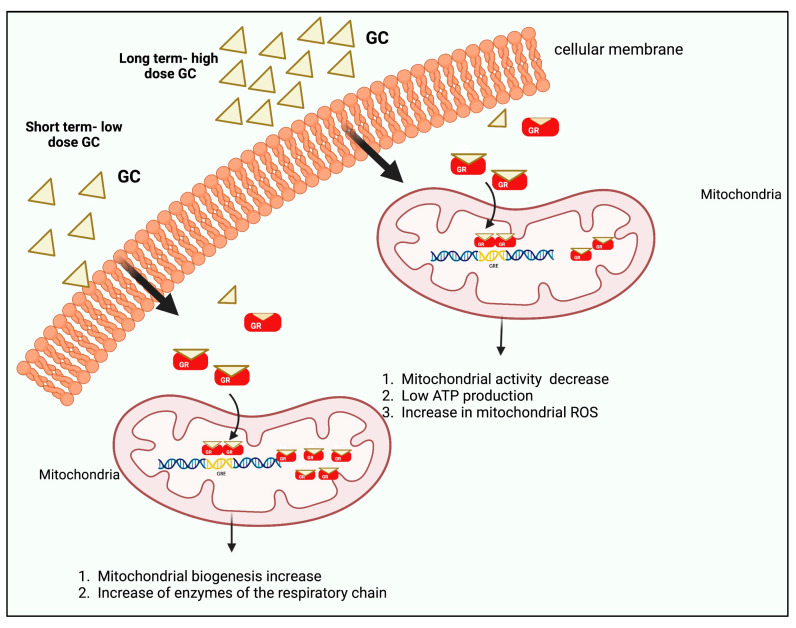
GCs exert biphasic effects on mitochondrial GR translocation. Short-term treatment with high or low doses of CORT/DEX induces the mitochondrial localization of GRs and leads to the increase of the GR/GC complex into mitochondria, thus enhancing mitochondrial function and cellular viability. Long-term treatment with high doses of CORT/DEX reduces the mitochondrial localization of GR and downregulates GR/GC complex in mitochondria, leading to mitochondrial dysfunction and apoptosis. Created by Biorender.

It has been reported that the GR can be localized within the mitochondria [57,58] and that some mitochondrial genes such as COX1, COX3, ND1, ND3, ND2, ND6, ATP6, ATP8, and Cytochrome b (CYTB) modify their expression upon exposure to DEX [59]. In addition, some of these genes, for example, COX1, have GRE sequences and can therefore be regulated at the transcriptional level [60]. It would be interesting to analyze the effect of DEX at low and high doses in ER-positive, progesterone receptor (PR)-positive, human epidermal growth factor receptor 2 (HER2)-positive breast cancer cells, and in TNBC (Figure 2).

## 6. Glucocorticoids and Cancer

For a long time, synthetic GCs such as DEX and prednisone have been used to treat different diseases, including cancer, due to their anti-inflammatory properties, and to reduce the side effects of cytotoxic drugs. GCs reduce vomiting, allergies, edema, and pain [4,47,61,62]. However, the beneficial role of GCs in epithelial cell cancer has been controversial. In colon, kidney, bladder, prostate, cervical, ovarian, and pancreatic cancers, GC treatment induces resistance to cytotoxic drugs or radiation therapies [48,63,64].

In recent years, several groups of researchers have demonstrated that stress hormones such as CORT, through their GRs, participate in cancer cell progression, specifically in breast cancer, because the presence of CORT is classified as an endocrine malignancy [65,66,67,68]. In immortalized MCF10A breast cell cultures, the addition of DEX reduces cell death and increases survival rate via a PI3K-independent mechanism, and overexpresses B-cell lymphoma 2 (BCL2) [69].

GCs can mediate breast cancer proliferation by inhibiting nuclear factor erythroid 2 (Nrf2). This factor regulates the transcription and activity of oxidative stress proteins such as glutathione transferase, quinone reductase, superoxide dismutase, and catalase, among others [70]. In breast cancer, the HPA axis is overactivated; increased CORT release or high GC concentrations induce Nrf2 inactivation via direct binding. Consequently, cells have a lower cellular defense system by repressing the transcription of oxidative stress proteins; this promotes cell proliferation in breast cancer and poor prognosis [71].

A low dose (10 nM) of DEX co-treatment has been shown to decrease the minimum inhibitory concentration (IC50) and increase the apoptotic effect of paclitaxel and paclitaxel-loaded nanoparticles in MCF7 cells. This modulatory effect has been associated with the ability of DEX to decrease the expression of the paclitaxel resistance gene (TRX1) and CYP3A4 mRNA level and increase the expression of the main paclitaxel metabolizing gene (CYP2C8) [72].

Pang et al. found that lower concentrations of DEX inhibit breast cancer tumor growth and metastasis, while higher concentrations may play an undesirable role in promoting breast cancer progression. Different doses of DEX may have the opposite effect on breast cancer development, progression, and metastasis [73].

Lin et al. showed that systemic GCs promote the survival of ER-negative breast cancer cells, leading to poor prognosis. In the cohort studied without chemotherapy, GC use was associated with the aggressive clinicopathologic features of breast cancer. In contrast, in the anthracycline cohort, multivariate analysis showed that GC use at each dose level was significantly associated with more prolonged breast cancer-specific survival (BCSS). We could conclude that concurrent use with GC improved the progression-free survival (PFS) and the overall survival of patients receiving adjuvant anthracycline-based chemotherapy for stage I–III breast cancer [74].

Furthermore, Abola et al. investigated in a phase III oncology clinical trial where treatment with higher relative toxicity has a higher PFS. This relationship between treatment toxicity and outcome efficacy may be of interest to patients who can tolerate some toxicity. Additionally, it has significance for research into individualized treatment using increased dosing, leading to higher side effects but potentially greater efficacy [75]. 

## 7. Glucocorticoids and Their Receptors in Breast Cancer

Breast cancer can be molecularly classified according to the expression of three markers in tumor cells: ER, PR, and HER2. The molecular classification was performed via immunohistochemistry, sequencing, and DNA microarray techniques [76,77,78,79,80].

The four subtypes of breast cancer are Luminal A, Luminal B, HER2+, and TNBC. The Luminal A (ER+, PR+, HER2−, Ki67 ≤ 20%) and Luminal B (ER+, PR+/−, HER2+/−, Ki67 > 20%) subtypes are characterized using ER and PR expression, are known as hormone receptor-positive (HRP), and are low-grade tumors with the best prognosis among all subtypes. HER2+ tumors (ER−, PR−, HER+) express only HER2, are generally high grade, and have an aggressive clinical course. TNBC or basal-like breast cancer (ER−, PR−, HER2-) does not express any of these markers and currently has the worst prognosis [47,81,82].

It has been reported that more than 60% of women with breast cancer have abnormal CORT levels during the day. Furthermore, the degree of mortality is higher in women with high CORT levels [83]. GC and GR participate with catecholamines in the biological response to stress. Diagnosis, treatment, fear of recurrence, functional disability, body image, and social interactions are a source of stress for cancer patients. Such stressful events experienced by the patient can significantly impact the patient’s health [84].

GCs are the most widely prescribed anti-inflammatory drugs for treating various immune disorders, inflammatory disorders, and cancer [24]. GCs have two main activities in gene transcription, transactivation, and transrepression. Transactivation is mainly mediated by the binding of GCs with their GRs to response sequences in DNA known as GREs. At the pharmacological level, they are associated with the side effects of GCs, such as glucose intolerance, diabetes mellitus, central obesity, osteoporosis, and muscle atrophy. On the other side, the transrepressor activity of GC is mainly associated with its beneficial therapeutic effects, such as the suppression of inflammation and immune activity, and the induction of apoptosis. A genome-wide study revealed that classical GRE and nGRE contribute to the transactivation and transrepression of glucocorticoid-responsive genes. Therefore, significant efforts have been made to produce GC with transrepression but without transactivation activity [85,86,87]).

Breast cancer gene 1 (BRCA1) is a tumor suppressor gene that is key to breast cancer development. GR without a ligand acts as a positive regulator of BRCA1. This beneficial effect is lost upon adding a ligand as CORT, which is a negative regulator of BRCA1 expression that increases the risk for breast cancer [22]. GR expression is essential for breast cancer prognosis and is associated with poor prognosis in ER-negative [88].

Ritter et al. demonstrated the ligand-independent role of GR in the activation of BRCA1 expression in mammary cells. The authors reported that GR regulates BRCA1 expression in a ligand-independent manner, with the intervention of the transcription factor guanine adenine-binding protein (GABP) transcription factor [89]. The heterogeneous expression of ER, GR, and PR in tumors indicates that treatments targeting hormone receptors will have non-uniform effects on breast cancer cells [90,91].

### Glucocorticoid Receptors in Triple Negative Breast Cancer

Recently, an association between high GR expression and chemotherapy resistance, and increased mortality in TNBC has been reported. Buoso et al. established evidence in cell lines from TNBC for the mechanism by which GR transcriptionally regulates a scaffolding protein with a GRE site (RACK), through the splicing factor SRSF3, demonstrating the participation of the latter in breast cancer cell migration and invasion. It was also established that this mechanism can be positively regulated by cortisol, which promotes an increase in SRSF3 gene expression levels in MDA-MB- 231 [92].

Another study suggested that GR signaling activation could induce breast cancer metastasis. Obradović et al. employed tumor transcriptional profiling in an NOD-scid-Il2rgnull (NSG) immunodeficient mouse xenograft model, to explore the signaling mechanisms involved in metastasis. Transcriptional profiling revealed that cancer cells clustered according to the site of metastasis, and pathway analysis indicated increased GR activity in metastasis. In addition, plasma levels of cortisol, corticosterone, and adrenocorticotropic hormone were higher in mice with metastasis than in healthy control mice. A proteomic analysis of GR-activated cells revealed increased levels of receptor tyrosine kinase-like orphan receptor 1 (ROR1) kinase related to GR activation. In vivo, ROR1 levels were higher in metastasis. A metastatic breast cancer database showed a significant positive correlation between ROR1 mRNA levels and a signature of GR activation. In vitro, the activation of GR by DEX increased the colonization capacity of multiple cell lines, and the in vivo activation of GR after tumor implantation enhanced metastasis formation and reduced overall survival. These data indicate that increased GR activity leads to the upregulation of ROR1, which promotes metastasis. It is of biomedical significance, as the results indicate that GR activation increases heterogeneity and metastasis. This suggests that caution should be used when administering GCs to patients receiving chemotherapy or patients with advanced disease, to prevent disease progression [93].

Prabhu et al. evaluated GR expression via immunohistochemistry in stromal cells and tumor-infiltrating lymphocytes. In TNBC, 54% showed GR expression in invasive tumors and 70% in tumor-infiltrating lymphocytes. It was known that GR expression in tumor-infiltrating lymphocytes is associated with immune-suppressive activity; however, it was observed that higher GR expression in immune cells was associated with better survival. It was even observed that in TNBC, tumors that are characterized by the absence of GR had higher lymph node metastasis. This indicates that GR expression in tumor-infiltrating lymphocytes and stromal cells is favorable in TNBC [94]. 

Mojica et al. analyzed the molecular mechanism of a GC-inducible and GC-regulated gene that has been implicated in cell cycle control, and it is the tumor suppressor gene ErbB Receptor Feed-back Inhibitor 1 (ERRFI1, also known as MIG6 or RALT) that functions as a negative feedback inhibitor of epidermal growth factor receptor signaling (EGFR), and it may mediate some of the effects of GCs in TNBC. They determined that there is a progressive loss of GC-dependent regulation that confers pro-tumorigenic effects via ERRFI1 in the metastatic TNBC cell model MDA-MB-231. This influences TNBC progression, and it may contribute to the unfavorable effects of GC therapy in TNBC. Thus, they conclude that GCs act as pro-oncogenic signals in TNBC, and that ERRFI1 plays divergent roles in TNBC progression [62].

GC signaling has also been shown to interact with the hypoxia-inducible factor (HIF) pathway to serve as a stress-sensing mechanism in TNBC. Regan et al. studied the crosstalk between GR signaling driven by CORT, the stress hormone, and physiological stress regulated by HIF. They found that HIF and GR co-assembled at the breast tumor kinase BRK (PTK6) promoter in response to hypoxia or GCs. They also found that the steroid receptor coactivator proline, glutamate, and leucine-rich protein 1 (PELP1) were induced in an HIF-dependent manner. Additionally, they discovered how PELP1 interacts with GR to activate BRK expression. Their findings linked cellular stress HIF and CORT signaling in TNBC, identifying the phospho-GR/HIF/PELP1 complex as a potential therapeutic target to limit Brk-driven progression and metastasis in TNBC patients [95].

Interestingly, the inhibitory effect of DEX at a concentration of 100 nM has been reported in a small population of the MCF7 tumor cell lines, termed the side population (SP), which has similar characteristics to stem cells. In this study, DEX was observed to decrease the growth of this population through the upregulation of ATP binding cassette transporter, subfamily G, member 2 (ABCG2) expression. This finding may suggest that DEX may be adjuvant in treating breast cancer by sensitizing cells to chemotherapy [96].

Consistent with these studies, it has been shown that there is a correlation between GR signaling pathways and metastasis. The GR through different signaling pathways can be used as a promising target to limit progression and metastasis in TNBC.

## 8. Glucocorticoids, Glucocorticoid Receptors, and the Treatment of Triple Negative Breast Cancer

TNBC has no effective targeted therapy in contrast to other breast cancer subtypes. It is characterized by the absence of ER and PR expression, and by HER2 amplification. Current treatments such as Tamoxifen, which is a selective estrogen receptor modulator (SERM), or Herceptin or Trastuzumab as an HER2 inhibitor, are not successful in this type of breast cancer, and so it is necessary to explore alternative drugs and therapies [97]. Patients with TNBC can only be treated with a combination of chemotherapy, radiotherapy, immunotherapy, and surgery. The intrinsic characteristics of the disease cause these patients to have the worst prognosis, with a high recurrence rate within three years and a high mortality rate of 38% [98,99].

In all breast cancer subtypes, GR is expressed to a variable degree. However, there is evidence that its mRNA is overexpressed in TNBC cell lines than in the other subtypes [100]. This overexpression of GR and specific GR-regulated genes is associated with shorter relapse-free survival (RFS) in TNBC patients, but with a longer RFS in receptor-positive breast patients [81].

Several side effects could occur after GC treatment. It is documented that a high expression of GR in TNBC is associated with chemoresistance and disease recurrence. Likewise, in ER-negative breast cancer, it is a marker of poor prognosis [101]. However, in ER-positive breast cancer, elevated GR expression is associated with improved overall survival and a better prognosis [102].

Wu et al. observed in MDA-MB-231 cells that GR induces the expression of multiple genes that promote cell survival and that include the SGK-1 and BCL family proteins. It is proposed that GC promotes MAPK dephosphorylation, and that SGK-1 and MAP kinase phosphatase-1 (MKP-1) are GR target genes that act together to inhibit MAPK phosphorylation, thereby decreasing the efficacy of chemotherapy-induced cell death [103].

However, Elkashif et al. found that in patients with TNBC, GR overexpression indicates a favorable response to anthracycline-based chemotherapy. Thus, the authors propose GR as a biomarker of first-line therapy and treatment response. The utility of GR as a biomarker is twofold, predicting which patients will respond well to anthracycline-based chemotherapy, as well as those who will respond poorly to taxane-based treatments. Using GC would reduce the costs and side effects, and improve therapy success [104].

Moreover, Conway et al. showed that GR and signal transducer and activator of transcription 3 (STAT3) are the main transcription factors that bind to the unmethylated and open chromatin regions of the genome of TNBC patient samples and cell lines. The authors determined that GR and STAT3 binding are essential factors, as together they can regulate genes such as EGFR and TGFB, promoting an aggressive type of breast cancer, and promoting proliferation, stemness, and EMT. They propose that the inhibition of both GR and STAT3 transcription factors may lead to an effective strategy for the treatment of TNBC [81].

In a xenograft study of MDA-MB-231, DEX administration was observed to reduce paclitaxel efficacy and increase tumor size. In addition, DEX was observed to induce the nuclear localization of GR and the overexpression of the anti-apoptotic gene MKP-1, and reduced the expression of apoptotic proteins such as Bid and TRAIL. It is concluded that GC promotes malignancy in breast cancer [105]. In addition, Lin et al. showed that the addition of GC to cancer cells increases the expression of SGK1, MKP-1, and IκBα with proliferation signaling [106].

There is controversial evidence for the use of GC in breast cancer; it was suggested that DEX has beneficial effects in ER-positive breast cancer by blocking cell cycle progression in the G0/G1 phase. However, ER-negative breast cancer, or TNBC has the opposite effect [107,108]. Furthermore, in the MCF7 breast cancer cell line, Karmakar et al. observed that GR directly binds to estrogen binding sites (EBS), inhibiting ER transcription and cell proliferation mediated by E2-ERα activity. This provides a basis for understanding the molecular mechanism of GR response in the different subtypes of breast cancer and to establish a basis for GC treatment in order to improve safety and effectiveness [109].

## 9. Glucocorticoids and Drug resistance in Triple Negative Breast Cancer

One of the main problems with chemotherapy is the development of resistance by the tumor cell. In vitro and in vivo studies suggest that DEX is an agent that modifies the sensitivity of tumor cells to the effects of chemotherapy. However, most publications refer to the fact that DEX inhibits antineoplastic-induced cell death [44,105], and other studies indicate that it may potentiate the effect of chemotherapy [64].

GR is involved in the transcriptional regulation of essential genes for tumor growth and metastasis in breast cancer [73,93]. Therefore, several research groups have focused on understanding the mechanisms that are implicated between GR and cell survival. One example is the GR–IRS-1 (insulin receptor substrate 1) axis, which is essential for regulating breast cancer cell survival, invasion, and metastasis [110].

The gradual loss of GC-dependent upregulation and the antitumor function of the tumor suppressor gene ERRFI1 may affect breast cancer progression and contribute to ineffective outcomes of GC therapy in TNBC [62].

Among the most widely used chemo agents for the treatment of TNBC is docetaxel, which uses polysorbate 80 as a vehicle because it is insoluble in water [111]. Unfortunately, this compound generates allergic reactions in breast cancer patients, so DEX is used to counteract such reactions. The group of Li et al. showed that DEX induces chemoresistance to docetaxel and cisplatin in TNBC by inducing Kruppel-like factor 5 (KLF5) in the HCC1937 and HCC1806 cell lines, as well as in a xenotransplant model. The authors found that DEX upregulates the expression of the transcription factor KLF5 at GR-dependent mRNA and protein levels. This promotes cell proliferation, survival, and tumorigenesis, resulting in chemoresistance in TNBC [112].

It is known that in ER-negative breast cancer patients, the genomic binding of GR to GRE is the principal mechanism for the regulation of genes that are associated with drug resistance and worst outcomes [92]. GR activates oncogenes, inhibits apoptosis, and blocks tumor suppressor genes in TNBC, leading to poor patient prognosis. The overexpression of GR is associated with increased mortality in patients with TNBC, and poor prognosis in ER-negative cancer. Furthermore, high GR expression is associated with chemoresistance and increased recurrence [113,114].

On the other side, it has been demonstrated that GR antagonism can help to sensitize TNBC in chemotherapy-induced cytotoxicity, so GR could be used as a biomarker in TNBC to improve diagnostics, prognostics, and therapy. Therefore, it is important to consider the complete GR activity signature in TNBC for patient stratification, to identify individuals who could benefit from therapies based on GR antagonism [92].

TNBC lacks effective targeted therapies, and consequently, cytotoxic chemotherapy offers the only systemic treatment option. The GR-mediated regulation of gene expression contributes to chemotherapy resistance. The activation of GR in TNBC may contribute to chemotherapy resistance in tumor cells following the activation of GR by endogenous cortisol [115].

## 10. The Role of the GR as a Biomarker in Breast Cancer Progression

GCs promote different effects according to the breast cancer subtype. GR is an important biomarker, as its high expression correlates with poor prognosis in ER-negative, and good prognosis in ER-positive breast cancer.

In ER-positive breast cancer, a high GR expression correlates with a better prognosis and a relapse-free survival outcome. Several in vitro experiments have demonstrated the ability of GCs to inhibit proliferation and to alter cell cycle progression. It has been observed that GR expression is repressed due to two distinct mechanisms: the methylation of its promoter on CpG islands, and its degradation in the proteasome. 

In ER-negative breast cancer, GR expression is associated with a poor prognosis, shorter survival, and an earlier relapse in the early stages. Specific DEX-induced GR target genes have been identified in the genome which is involved in tumor cell survival and chemotherapy resistance, the EMT, chromatin remodeling, and epithelial cell–inflammatory cell interactions, suggesting GR involvement in aggressive behavior. DEX-bound GR has been shown to bind to the GRE of pro-tumorigenic genes that drive chemotherapy resistance and TNBC progression [6].

## 11. Future Perspectives

This review discusses the role of GCs and their GRs in breast cancer, highlighting unresolved controversies regarding the use of GCs in cancer treatment, with a specific focus on TNBC. In this medical necessity, many aspects remain to be discovered. Further studies are important for understanding the impact of breast cancer progression in the different subtypes, as their action is different in each of them. TNBC is one of the most aggressive subtypes of breast cancer, in which the lack of hormone receptors complicates treatment. Therefore, it is important to analyze its effects at the genomic and non-genomic levels. 

Our proposal is based on a mitochondrial model in which we can measure the activation of mitochondria and EMT with high and low doses of GCs over short and long periods of time. Moreover, this allows an analysis of the expression changes in the transcriptome at the gene expression level that are associated with the EMT process and mitochondrial activation, to determine whether these genes correspond to a metastatic model, in order to find new diagnostic and therapeutic targets. 

## 12. Conclusions

GCs are pleiotropic molecules that are essential in the stress response, and they have anti-inflammatory, tumor development, and antiemetic properties that aid in the patient’s response to chemotherapy. Despite their serious side effects in therapies, they remain among the most prescribed drugs worldwide. DEX can act as a double-edged sword when it is used at different doses during breast cancer progression and metastasis [73]. However, the role of GC in tumor progression is a question that remains unresolved.

The modulatory capacity possessed by GC in upregulating or downregulating resistance genes and modulatory genes to chemotherapy may be an excellent tool for the development of improved therapies in TNBC [72]. In addition, it is well known that TNBC cells express GRs to a variable degree, making GRs a potential therapeutic target.

It has been proposed that chemotherapy would be most effective if administered at the time of day when circulating GC levels are at their lowest; otherwise, chemoresistance has been observed [43]. Therefore, further studies would be necessary to establish whether the circadian rhythm of circulating GC levels can be employed in order to facilitate advances in breast cancer therapy.

Several fields remain to be studied in breast cancer and in the action of GC. This includes the effect on mitochondrial function, where it has been found that under normal conditions, the GR is present and can activate the transcription of mitochondrial genes [116]. However, its role in breast cancer, mainly in TNBC, is unknown. 

Different types of cancer, differential levels of GR, the dose of GC administered, and even the activation of other nuclear hormone receptors, such as PR or ER, must be considered. Understanding the mechanisms by which GR regulates transcription and their downstream influences on ER and PR activity is critical to improving the efficient clinical targeting of hormone signaling for breast cancer treatment [117]. However, more than experimental models may be required to predict therapeutic outcomes accurately.

## Figures and Tables

**Table 1:** Main post-translational modifications of GRs.

Region	Modification	Residues	Aftermath	Ref.
Amino-terminal domain	Phosphorylation in 11 serine sites	T8, S45, S113, S134, S141, S203, S211, S226, S234, S267, S404	Affect target gene expression, nuclear export, binding with other proteins, conformational changes, and degradation	[4]
	SUMOylation in two residues	K277, K293		
	Ubiquitination in one site	K419		
Ligand-binding domain	Oxidation	C481	General activity	[21]
Hinge region	Four sites to acetylation	K480, K492, K494, K495	Binding to DNA	[22]
Ligand-binding domain	One site for SUMOylation	L703	Binding to other molecules	[23]

**Table 2:** Genomic and non-genomic effects of glucocorticoids.

Modality	Effects	Examples	Ref.
GC interacts with the cellular membrane	Influences membrane fluidity and composition and leads to regulation of signal-transducer/effector systems.	Membrane stabilizing effects of GCs in rat liver.	[29]
GC interacts with GR in the cellular membrane	GC binds a GR localized in the plasma membrane, leading to the regulation of signal-transducer/effector systems.	Membrane glucocorticoid receptors are expressed in T lymphocytes.	[30]
GC binds cytoplasmic GR	GC binds cytoplasmatic GR but does not travel to the nucleus.	Dexamethasone enhances the activation of endothelial NO synthase in the heart.	[31]
GC binds to other membrane receptors	GC binds a putative pertussis toxin-sensitive inhibitory G-protein coupled receptor.	These non-genomic mechanisms are involved in the glucocorticoid-negative regulation of ACTH expression, and a pertussis toxin-sensitive GTP-binding protein participates.	[32]
Mitochondrial GC	GC is introduced into the mitochondria and binds to mitochondrial GRE elements, or binds to mitochondrial proteins, affecting their functions.	In lung and during hepatic inflammation.	[33]
